# Effect of H_2_O Molecule Adsorption on the Electronic Structure and Optical Properties of the CsI(Na) Crystal

**DOI:** 10.3390/ma14071720

**Published:** 2021-03-31

**Authors:** Fang Liu, Hao Zheng, Tianze Jiang, Bin Liu, Jianming Zhang, Hang Yuan, Fengcheng Liu, Xiaoxue Fan, Xiaoping Ouyang

**Affiliations:** 1 Beijing Key Laboratory of Passive Safety Technology for Nuclear Energy, School of Nuclear Science and Engineering, North China Electric Power University, Beijing 102206, China; liuf@ncepu.edu.cn (F.L.); Jiangtz@ncepu.edu.cn (T.J.); NEzjm@ncepu.edu.cn (J.Z.); fcliu@ncepu.edu.cn (F.L.); Fanxx@ncepu.edu.cn (X.F.); 2 China Nuclear Power Engineering Co., Ltd., Beijing 100840, China; zhenghao@cnpe.cc; 3 Science and Technology on Reactor System Design Technology Laboratory, Chengdu 610213, China; yuanhang_npic@163.com; 4 Radiation Detection Research Center, Northwest Institute of Nuclear Technology, Xi’an 710024, China; oyxp2003@aliyun.com; 5 Department of Engineering Physics, Tsinghua University, Beijing 100084, China

**Keywords:** H_2_O molecule adsorption, first principle, CsI(Na) crystal surface, optical property

## Abstract

We investigated H_2_O molecule adsorption that had an effect on the luminescence properties of the CsI(Na) crystal using experiments and first-principle calculations. We measured the emission spectra of the CsI(Na) crystal at different exposure times under gamma ray excitation. The experimental results showed that the energy resolution of the CsI(Na) crystal was worse when the crystal surface adsorbed more H_2_O molecules, and the crystal surface deliquescence decreased the luminescence efficiency of the CsI(Na) crystal. We studied the band structure, density of states, and optical properties changes caused by H_2_O molecule adsorption on the CsI(Na) (010) surface. The generalized gradient approximation (GGA) was used to describe the exchange and correlation potential between the electrons. Our calculation results showed that the band gap width of the CsI(Na) (010) surface decreased after adsorbing H_2_O molecules, while three new peaks appeared in the valence band, and the absorption coefficient decreased from 90,000 cm^−1^ to 65,000 cm^−1^, and the reflection coefficient decreased from 0.195 to 0.105. Further, the absorption coefficient was reduced by at least 25% because of H_2_O molecule adsorption, which led to the luminescence degradation of the CsI(Na) crystal.

## 1. Introduction

Cesium iodide doped with sodium (CsI(Na)) crystal has been used as a charged particle detection material since the late 1960s [1]. Recently, CsI(Na) crystal has been widely used in γ ray spectrometry [2] due to its high emission efficiency and strong stopping power. However, the Na^+^ ions in CsI(Na) crystal adsorbing H_2_O molecules easily lead to luminescence efficiency decrease of Na^+^ ions as emission centers; this phenomenon is observed when the CsI(Na) is not appropriately encapsulated [3]. Deliquescence-induced Na^+^ ion reduction occurs in the surface layer, and if the thickness of the surface layer is more than 1 μm, the α particles (with energy less than 100 keV) and β rays (with energy less than 10 keV) are unable to touch the Na^+^ ion emission centers, but γ rays can pass through the deliquescent CsI(Na) surface layer. The time-dependent photoluminescence and radioluminescence of CsI(Na) single crystals exposed to 50% and 75% relative humidity were investigated by other researchers; the experimental results [4] show that moisture condensation contributed to the “dead” layer development, and the “dead” layer activation concentration was the below scintillation threshold due to the outward diffusion of Na, which led to scintillation degradation of CsI(Na) crystals if exposed to the air. Our pulse height spectrum measurement results for CsI(Na) samples exposed to 85% relative humidity indicate the scintillation decreased with a longer exposure time. Our results also indicate that the CsI(Na) crystal surface absorbing H_2_O molecules changed the state and structure of the crystal surface and affected the scintillation characteristics, especially those of the film crystal. Studies [5,6] on Na-activated CsI detectors indicated that the detection efficiency of these crystals degraded rapidly under high humidity. The influence of air exposure on the structure, resistivity, and infrared transmittance of the CsI film were investigated by scanning electron microscopy and X-ray diffraction (XRD) [7]; the XRD results indicated the formation of a (110/220) texture when exposed to ambient air and the relaxation of tensile stress during recrystallization. CsI crystal is the host material of the CsI(Na) crystal; the electronic structure, electronic density of states, and optical properties of the CsI and CsI:Ag have been studied using a first-principle calculation based on density functional theory (DFT) [8].

All these facts result in the surface structure changes of the CsI(Na) crystals and the degradation of the CsI(Na) surface scintillation after the CsI(Na) crystal surface absorbs H_2_O molecules. The band structure, density of states, and optical properties affect the scintillation performance. In this paper, we measure the pulse height spectra of the CsI(Na) crystal with different air exposure times and different air humidity environments under γ ray excitation and investigate the effect of the CsI(Na) crystal surface absorbing H_2_O molecules on the scintillation degradation. We use Cambridge sequential total energy package (CASTEP) software to calculate band gap, density of states, and optical properties with the aim of determining the key factors leading to CsI(Na) crystal surface scintillation degradation after the absorption of H_2_O molecules.

## 2. Materials and Methods 

### 2.1. Theoretical Method

**Figure 1:**
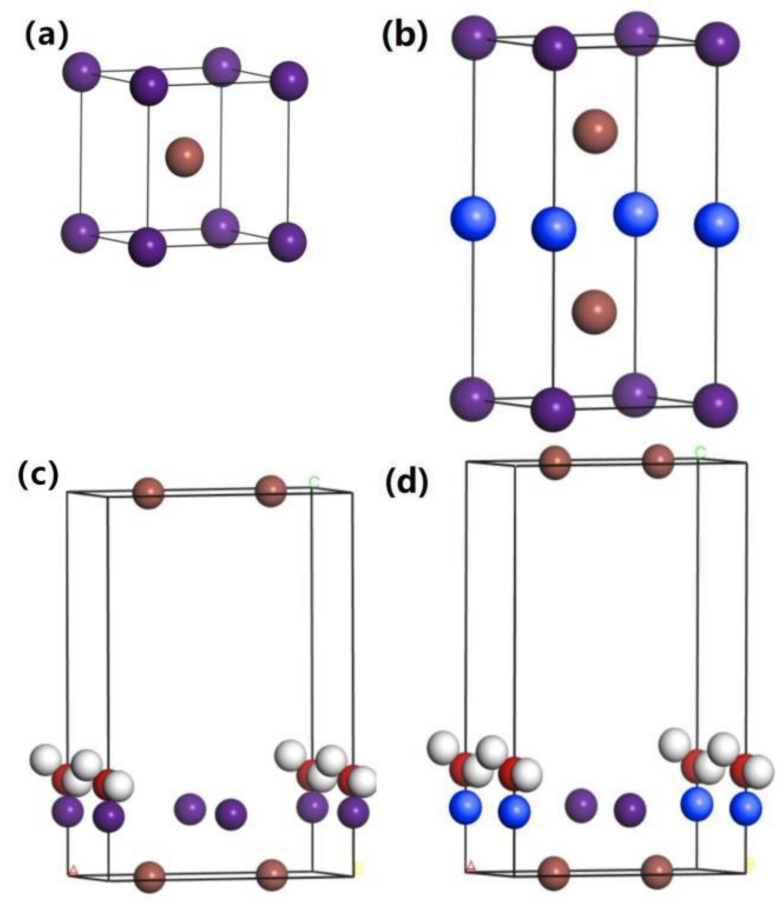
The computing models of the CsI crystals (**a**), CsI(Na) crystals (**b**), H_2_O molecules adsorbed on the CsI (010) surface (**c**) and H_2_O molecules adsorbed on the CsI(Na) (010) surface (**d**) (brown balls, purple balls, and blue balls represent I^−^, Cs^+^, and Na^+^ ions, respectively).

The crystal structure of the CsI is a cubic crystal system, and its space group is Pm3m. Figure 1a,b show the computing models of the CsI crystals and CsI(Na) crystals. Figure 1a shows a 1 × 2 × 1 supercell of the CsI crystal, and in each CsI crystal, cell eight cesium atoms are located in the cubic vertex angle and one iodine atom is located in the body center. In the CsI(Na) crystal, one cesium atom in the supercell is replaced by a sodium atom as shown in Figure 1b. The 50% substitution concentration we used corresponds to the microscopic doping points. The extreme model used in the calculation increases the difference between the two crystals. The H_2_O molecules are adsorbed on the CsI (010) surface and CsI(Na) (010) surface; those structural models are shown in Figure 1c,d. H_2_O molecules are adsorbed in addition to Na^+^ because the hygroscopicity of the CsI(Na) crystal is caused by the doping of Na^+^ ions that easily adsorb H_2_O molecules.

The calculations performed in this investigation were completed by the Cambridge Sequential Total Energy Package (CASTEP) loaded on Materials Studio 8.0 software. CASTEP is an ab initio quantum mechanics package based on density functional theory (DFT), it was originally developed by Cambridge University, UK. Instead of ion potential, the planar wave pseudopotential method is used to expand the electronic wave function. The exchange correlation between electrons is described by local density approximation (LDA) or generalized gradient approximation (GGA). GGA is adequate for large density gradient systems; the optimized configuration is more consistent with reality. The GGA approximate ultra-soft method in the form of PBE (Perdew Burke Ernzerhof) is used to optimize the crystal structure of the CsI and CsI(Na) crystals. All the initial lattice parameters used for calculations came from experiments.

GGA PBE tends to underestimate the band gaps, so we chose the Heyd–Scuseria–Ernzerh (HSE) [9,10,11] hybrid function for more accurate property calculations. We used HSE06, because it is more suitable than HSE03 for calculating the band gaps of semiconductors [12]. The kinetic energy cutoff is 300 eV, which is optimized by balancing the reliability of results and the amount of computation. Space representation is reciprocal, self-consistent field (SCF) tolerance is 1.0 × 10^−6^ eV/atom, and k sampling is a 5 × 5 × 5 k-point mesh in the Brillouin zone. We confirmed the optimal atomic positions when the following criteria were satisfied: 1. the maximum force on the atomic positions is smaller than 0.05 eV/nm; 2. the maximum change in the energy per atom is smaller than 1.0 × 10^−5^ eV; 3. the maximum displacement is smaller than 0.001 Å; and 4. the maximum stress on the crystal is smaller than 0.02 GPa. We calculated all the properties based on the crystal structure optimization.

### 2.2. Experimental Setup

**Figure 2:**
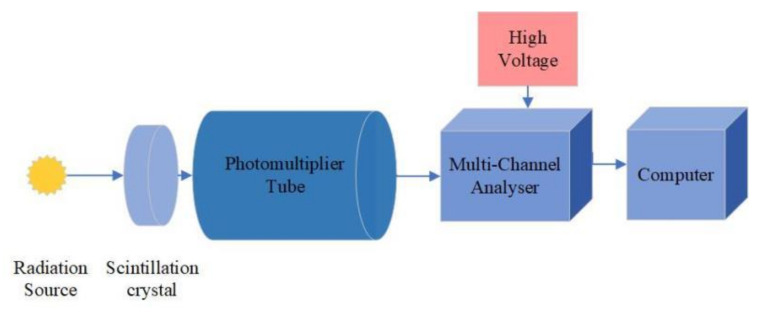
Experimental Setup.

In our experiments, we used commercial CsI(Na) crystal materials (HAMAMATSU, Hamamatsu, Japan) with 5% mol Na-ion doping concentration and measured the γ ray-excited emission spectra of crystals with the setup shown in Figure 2. The radionuclides ^137^Cs- and ^60^Co-radiate γ rays with energies 662 keV, 1.17 MeV, and 1.33 MeV. The CsI(Na) crystals are coupled to a photomultiplier (PMT) using silicone oil as a coupling agent. The emission peak wavelength of the CsI(Na) crystals is 420 nm, so we chose the photomultiplier (Hamamatsu, CR-105, response wavelength range 300–650 nm) with a 420 nm peak response wavelength, which matched the CsI(Na) crystals well. The photomultiplier tube coupled to the crystals converts the input light to electrical signals, which are then amplified and processed in the tube socket and multichannel analysis unit (CANBERRA, Bois Mouton, France, OSPREY-PKG+). We used the spectrum analysis software (CANBERRA, Bois Mouton, France, Genie 2000) to adjust the HV and gain of PMT on the computer to acquire and analyze the detector spectrum. The crystals and PMT were assembled in a lead box for light shading and ray shielding. Figure 2 indicates the flowchart diagram of the experimental setup.

## 3. Data Analysis

### 3.1. Energy Spectrum Measurement in Different Relative Humidity Environments

**Figure 3:**
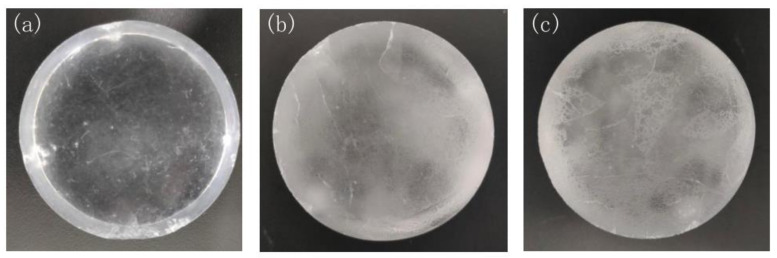
CsI(Na) crystal (size: Φ50 mm × 29 mm) (**a**–**c**) exposed to 85% relative humidity air for 0, 8, and 10 h respectively.

**Figure 4:**
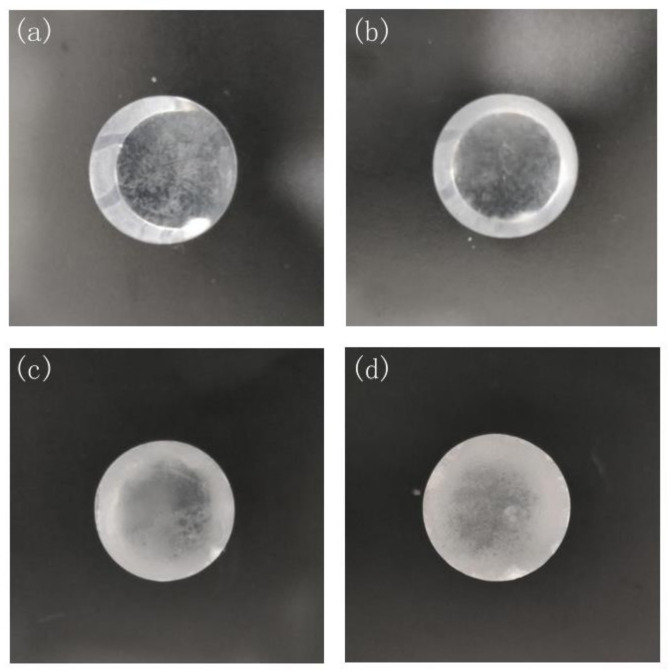
CsI(Na) crystal (size: Φ25 mm × 50 mm) (**a**–**d**) exposed to 85% relative humidity air for 0, 2, 4, and 12 h respectively.

In order to investigate the CsI(Na) crystal scintillation degradation caused by the crystal surface adsorbing H_2_O molecules, we exposed CsI(Na) crystal to 25% relative humidity air at 15 °C temperature and 85% relative humidity air with different exposure time. Figure 3 and Figure 4 show the CsI(Na) crystal samples that were exposed for different times; we can see that the CsI(Na) crystal surface becomes more blurred with a longer exposure period; these blurred layers on the crystal surface, called “dead ” or “inactive” layers [6,13] on the surface of deteriorated detectors, were indirectly illustrated by the decrease in the scintillation performance. We measured the γ ray-excited emission spectra of crystals using the experimental setup in Figure 2 to study the effect caused by the “dead” layer under ^137^Cs-662 keV γ ray excitation.

The luminescence efficiency variation of the CsI(Na) scintillators and the luminescence linearity of the crystal under different energy ray excitation tend to affect the detection performance in the actual detection process, and the energy resolution reflects the result of the combined effects of these two factors. Energy resolution characterizes the ability of nuclear radiation detectors to distinguish the full-energy Gaussian peak of γ photons with similar energy and is an important index of a detector. For incident particles with different energy, the smaller the energy resolution value of the detector, the lower the overlap degree of full-energy Gaussian peaks, which indicates the detector has a better ability to distinguish rays in complex multi-radioactive radiation field. 

In our experiments, the energy resolution was obtained for the 662 keV photoelectric peak of ^137^Cs, and we calibrated the channels with the ^60^Co γ-ray (1.17 MeV and 1.33 MeV) energy spectrum measured simultaneously. The measurement time of each energy spectrum was 1000 s. The formula used for the energy resolution was FWHM/E.

In our experiments, we used two different sizes of CsI(Na) crystals, Φ50 mm × 29 mm and Φ25 mm × 50 mm. We performed the measurements at room temperature. Firstly, we measured the energy resolution of the CsI(Na) crystal (Φ25 mm × 50 mm) at 25% relative humidity. As can be seen from Table 1, the energy resolution remained at about 11% and fluctuated slightly under low humidity, and the energy resolution did not change distinctly.

**Figure 5:**
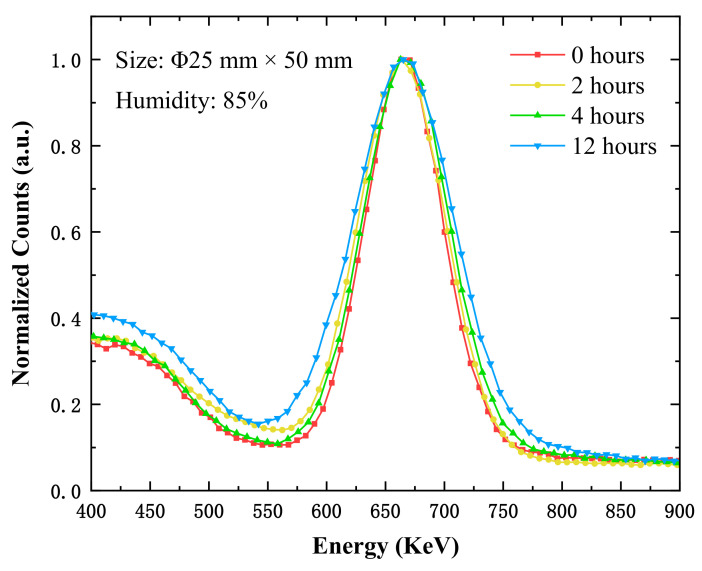
The energy spectra of the CsI(Na) crystal (size: Φ25 mm × 50 mm) with different exposure times at 85% relative humidity under ^137^Cs-662 keV γ ray excitation.

**Figure 6:**
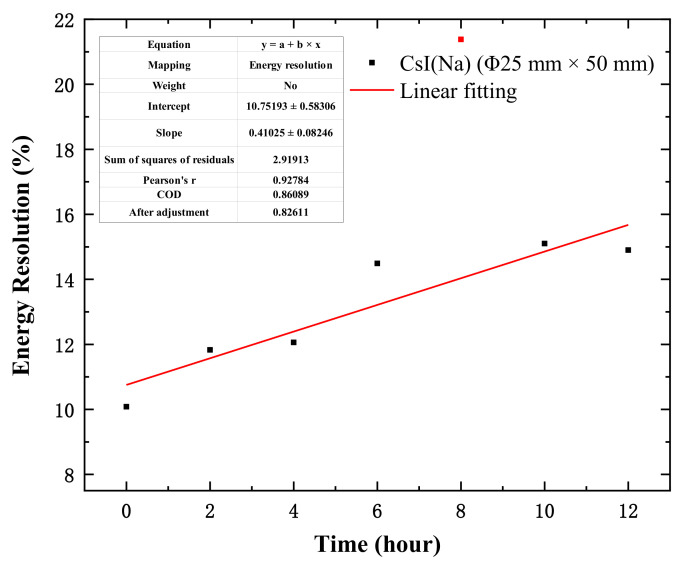
The energy resolution of the CsI(Na) crystal (size: Φ25 mm × 50 mm) with different exposure times at 85% relative humidity under ^137^Cs-662 keV γ ray excitation.

However, when the CsI(Na) crystal (Φ25 mm × 50 mm) was placed at 85% relative humidity, the experimental results in Figure 5 showed that the energy resolutions were 10.08%, 11.83%, 12.06%, and 14.90% when the exposure periods were 0 h, 2 h, 4 h, and 12 h, respectively. It can be seen that the CsI(Na) crystal (Φ25 mm × 50 mm) had better energy resolution at the beginning of deliquescence, and the energy resolution of the crystal deteriorated more obviously after a long period of exposure, shown in Figure 6 at 85% relative humidity.

**Figure 7:**
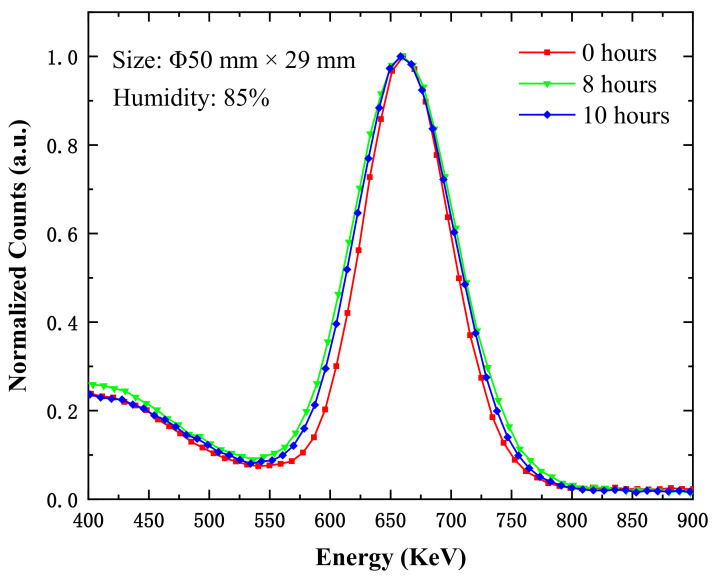
The energy spectra of the CsI(Na) crystal (size: Φ50 mm × 29 mm) with different exposure times at 85% relative humidity under ^137^Cs-662 keV γ ray excitation.

We measured the energy resolution of another CsI(Na) crystal with a size of Φ50 mm × 29 mm at 85% relative humidity; the energy resolutions were 12.90%, 14.22%, and 14.19% when the exposure periods were 0 h, 8 h, and 10 h, respectively. The experiment results show the energy resolution worsened with a longer exposure time; this worsening trend was the same as that of the CsI(Na) crystal with the size of Φ25 mm × 50 mm. The scintillation performance of the CsI(Na) crystal decreasing monotonically was reported in the literature [4] (see Figure 2), particularly under higher humidity conditions (RH > 50%) and with longer exposure times. When the CsI(Na) crystal surface absorbed H_2_O molecules, the formed “dead” layer affected the luminescence efficiency, which broadened the spectral curves in Figure 5 and Figure 7. The larger the energy resolution, the worse the scintillation performance of the crystal. The broadening of spectral curves was due to the H_2_O molecule adsorption caused by the Na diffusion away from the crystal, which left behind “dead “regions with an activator (Na) concentration below the lowest limit for scintillation (0.01%) [14].

### 3.2. Band Structure and Electronic Density of States

We optimized the geometry of the CsI and CsI(Na) unit cells to study the electronic structure and optical properties. The optimized lattice parameters for the supercells of the CsI and CsI(Na) were a_0_ = b_0_ = c_0_ = 4.827 Å and a_0_ = b_0_ = c_0_ = 4.503 Å, and the angles for all crystals were α = β = γ = 90°. These lattice parameters of the CsI crystal were in good agreement with the results in reference [15], while the lattice parameters of the CsI(Na) crystal were smaller due to doping.

In the model, the optimized O–H key in H_2_O molecules was about 0.973 Å, the O–H bond angle was 103.946 degrees, the O–H bond length of gaseous water molecules was 0.97 degrees, and the bond angle was 104 degrees. The surfaces of the CsI (010) and CsI(Na) (010) were optimized geometrically, and it was found that the surface atoms had varying degrees of relaxation. We used a molecule-in-a-box type of calculation to calculate the energy of a single water molecule. The dimensions of the box were a_0_ = b_0_ = c_0_ = 4.827 Å, the same as the lattice parameters of the CsI crystal, and we set a vacuum layer with a size 1.5 times the lattice around the single water molecule to avoid periodic interactions between that molecules. The results for geometric optimization of supercell and gaseous H_2_O molecules were identical to the theoretical values, which indicates that the selected model is appropriate and the calculation method is feasible [16,17].

The simulation models of H_2_O molecules adsorbing on the CsI and CsI(Na) surface are shown in Figure 1c,d. The adsorption properties of H_2_O molecules on the surface of the CsI (010) can be defined by the Formula (1) [18]:

(1) $$\Delta E_{ads}=E_{h_{2}o+surf}-E_{h_{2}o}-E_{surf}$$

where $E_{ads}$ is the adsorption energy those of CsI and CsI(Na) are shown in Table 2, $E_{h_{2}o+surf}$ is the total energy of the system when a single H_2_O molecule is adsorbed on the surface of the CsI (010) or CsI(Na) (010), $E_{h_{2}o}$ is the energy of a single gaseous H_2_O molecule, and $E_{surf}$ is the energy of the CsI (010) surface or CsI (010) (Na) surface.

The calculated results of two models in Figure 1c,d show that physical adsorption occurred on the surface. However, the adsorption energy of CsI (010) was positive, indicating that the surface of CsI (010) did not adsorb H_2_O molecules. This phenomenon is consistent with the theory that the CsI (010) surface has no deliquescent properties. However, on the CsI(Na) (010) surface that adsorbed H_2_O molecules, the bond length R_O-H_ and bond angle A_H-O-H_ of the molecules increased, which indicates that the interatomic force of H_2_O molecules decreased while the activity increased.

CsI(Na) crystal is a luminescence efficient scintillation crystal for radiation detection. When an electron in scintillator atoms receives energy from the incident particles which deposit larger energy than its forbidden band width, the electron is excited to the conduction band from the forbidden band. Then, it returns to the ground state and emits photons in the process of deexcitation. Therefore, the band structure and the density of state distribution are closely related to the characteristics of the scintillator after it is stimulated. We calculated the band structure and state density of the CsI and CsI(Na) crystals based on the geometric optimization model.

**Figure 8:**
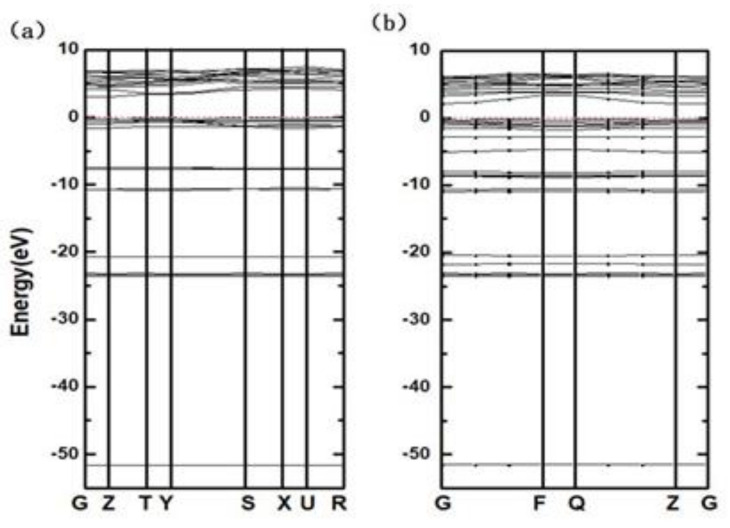
Band structures of the CsI(Na) (010) surface (**a**) and the CsI(Na) (010) surface adsorbing H_2_O molecules (**b**).

From Figure 8, it is observed that the calculated band width of the CsI(Na) is 2.7 eV, which is consistent with the calculated results in Zhao Qiang’s work [6], and the band width of the CsI(Na) (010) surface is 2.99 eV, which decreased to 2.118 eV after the CsI(Na) (010) surface adsorbed H_2_O molecules. In other words, H_2_O molecular adsorption influences the band width and the electron density of state distribution, and that in turn affects the electron excitation process.

**Figure 9:**
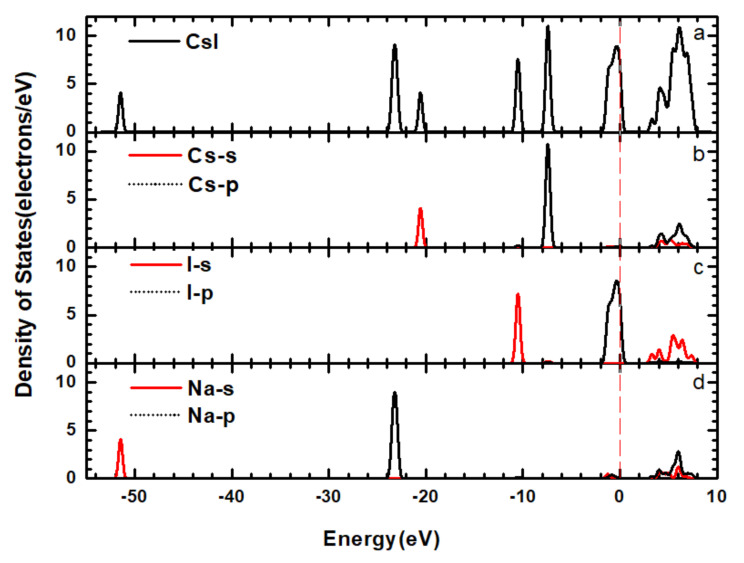
(Color online) TDOS (**a**)and PDOS (**b**–**d**) of the CsI(Na) (010) surface without H_2_O molecule adsorption.

**Figure 10:**
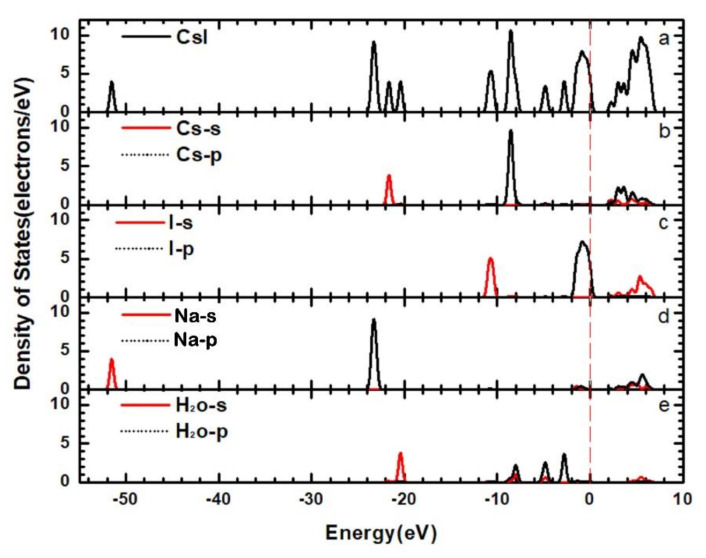
(Color online) TDOS (**a**) and PDOS (**b**–**e**) of the CsI(Na) (010) surface adsorbing H_2_O molecules.

In order to further study the formation of band structures, we calculated the electronic densities of states of the CsI(Na) (010) surface, which can explain the cause of band structure changes in detail. Figure 9 and Figure 10 show the total density of states (TDOS) and the partial densities of states (PDOS) for the CsI(Na) (010) surface and of the adsorbing H_2_O molecules. As we can see from the TDOS of the CsI(Na) crystal in Figure 9a, the conduction band was distributed between 2.5~8.5 eV and six peaks in the valence band (−51.5 eV, −23 eV, −20 eV, −10 eV, −7 eV and 1 eV). Among them, the peak value at −1 eV was wide, and its distribution range was between −2.5~0.5 eV. However, the PDOS distribution diagrams in Figure 10b–e indicate that the conduction band of the CsI(Na) crystal was mainly composed of the 5S orbit of I atoms, the 5S and 5p orbits of Cs atoms, and the 2S and 2p orbits of Na atoms. The two peaks near the Fermi level in the valence band (−7 eV and −1 eV) were composed of Cs’s 5p orbits and I’s 5p orbits. By comparing the TDOS in Figure 9 and Figure 10, it can be seen that the width of conduction band of the CsI(Na) (010) surface increased after adsorbing H_2_O molecules, and small peaks appeared at −3 eV, −5 eV, and −21 eV; the conduction band and the valence band increased some exciton bands near the Fermi level. Through PDOS analysis, we can see that the conduction band was composed of I’s 5S orbit, Cs’s 5S and 5p, and 2S and 2p orbits of Na. In the valence band, the three new peaks induced by H_2_O molecules near the Fermi level contributed to the TDOS changes in the CsI(Na) (010) surface that adsorbed H_2_O molecules.

### 3.3. Optical Properties

**Figure 11:**
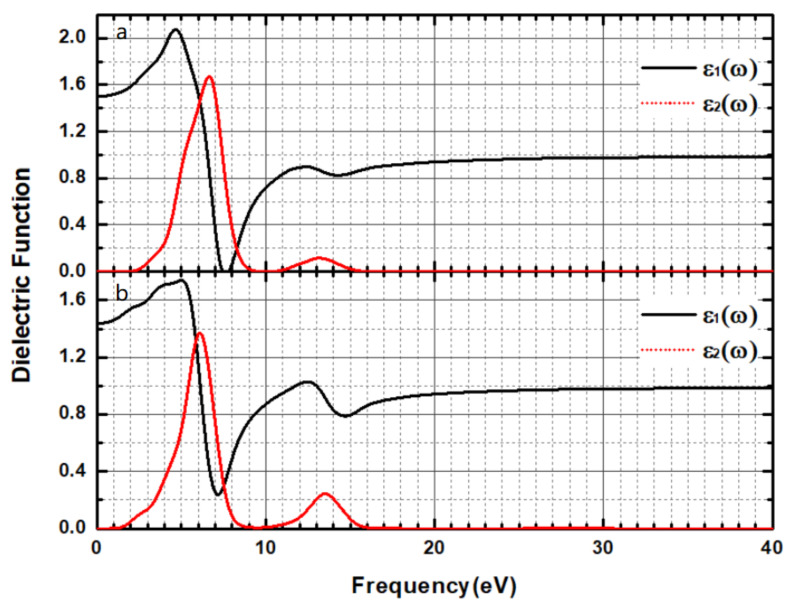
(Color online) Dielectric Function of the CsI(Na) (010) surface (**a**) and that of the CsI(Na) (010) surface adsorbing H_2_O molecules (**b**).

Figure 11 shows the real and imaginary parts of the dielectric function of the CsI(Na) (010) surface. Figure 11 shows that the absorption edge of the ε_2_ parameter for the CsI(Na) (010) surface starts from 2.5 eV, but starts from 1.5 eV after adsorbing H_2_O molecules, and their optical band gaps were 2.9 eV and 2.1 eV, respectively. These results are in good agreement with the calculated band structure. The first peak value before adsorption was about 6.75 eV and after absorption peak was located at 14 eV. It is worth noting that the energy difference between the 6.75 eV peaks and 14 eV peaks was 7.25 eV. After adsorption, the CsI(Na) (010) surface had two absorption peaks at 6.1 eV and 13.75 eV.

**Figure 12:**
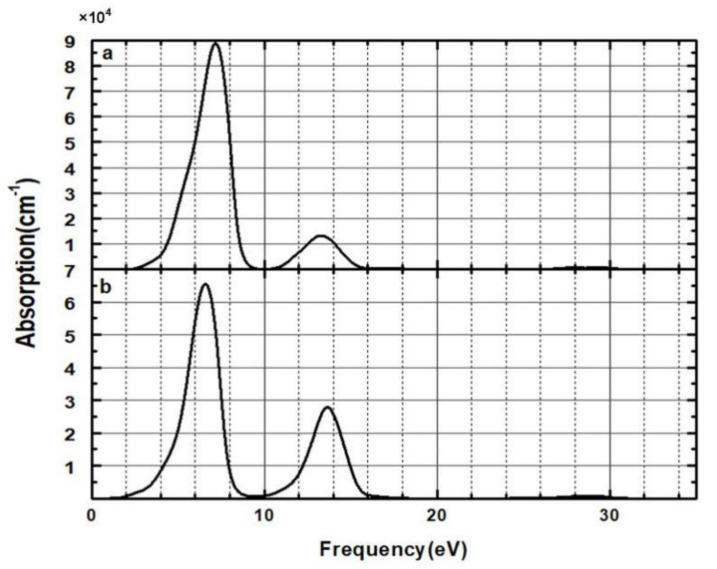
Absorption coefficient of the CsI(Na) (010) surface (**a**) and that of the CsI(Na) (010) surface adsorbing H_2_O molecules (**b**).

**Figure 13:**
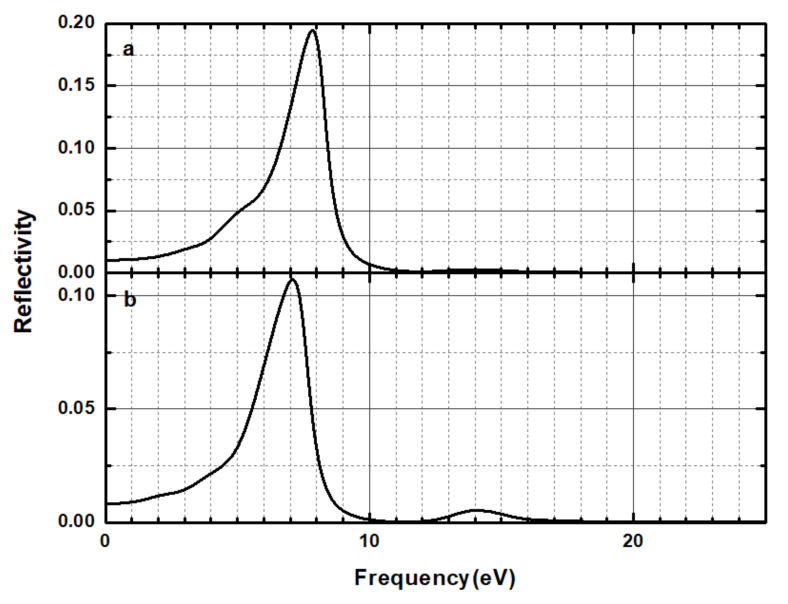
Reflectivity of the CsI(Na) (010) surface (**a**) and that of the CsI(Na) (010) surface adsorbing H_2_O molecules (**b**).

The absorption coefficient of a material indicates the light intensity attenuation percentage of the light wave propagating in that medium. In this paper, the calculated absorption coefficient of the CsI(Na) (010) surface that of the CsI(Na) (010) surface adsorbing H_2_O molecules were shown in Figure 12. In the range of photon energy less than 2.5 eV and 1.5 eV, the absorption coefficient of the CsI(Na) (010) surface and that of the CsI(Na) (010) surface adsorbing H_2_O molecules were zero. This shows that the relative energy of these photons is transparent. The absorption coefficient of the CsI(Na) (010) surface was 90,000 cm^−1^, but decreased to 66,000 cm^−1^ after adsorbing H_2_O molecules. The movement of the low energy end caused the electronic transition to be accomplished by only absorbing smaller energy, which eventually led to the tendency of the absorption spectrum to be moved to the low energy end (red shift). We can see that the H_2_O molecular adsorption was greatly reduced by the photon absorption of the CsI(Na) surface, which resulted in the scintillator detection efficiency decrease. Our result is consistent with the experimental results. For 500 nm thick CsI crystal, when it is under long term exposure (24 h), the absorption peak broadens with the band center shifting toward lower wave numbers [19]. The calculated reflection coefficients are shown in Figure 13. When the H_2_O molecules were adsorbed on the CsI(Na) (010) surface, the red shift of the reflection coefficient peak was observed, and the reflection of the photon was weak; the reflection coefficient decreased from 0.195 to 0.105. From this point of view, the adsorption of H_2_O molecules decreased the luminous efficiency of the CsI(Na) crystal.

## 4. Conclusions

In order to explain how adsorbing H_2_O molecules on the CsI(Na) (010) surface can affect the luminescence performance of the CsI(Na) crystal and discuss which factors induce crystal property changes resulting in the luminescence reduction, we measured the gamma ray spectra of the CsI(Na) crystal under different exposure conditions to investigate the luminescence efficiency changes after the adsorption of H_2_O molecules on the CsI(Na) (010) surface and optimized the structure of the CsI(Na) (010) surface to study the band structure, density of states, and optical properties using a first-principle calculation method based on DFT. The experimental results show that the energy resolution worsened with increasing crystal exposure time. This indicates that H_2_O molecules adsorbing on the CsI(Na) (010) surface caused the luminescence degradation. The calculation results reveal the changes in the band structure and optical properties of the CsI(Na) (010) surface because of H_2_O molecular adsorption. Our calculation results verify the experimental results and explain the scintillation degradation of CsI(Na) and highlight the importance of surface preparation for CsI(Na) crystal detectors. It is crucial to explore the luminescence efficiency effect caused by H_2_O molecular adsorption, especially for crystals that readily experience deliquescence such as NaI(Tl), LaBr3(Ce), and SrI(Eu) crystals.

## Figures and Tables

**Table 1:** The energy resolution of the CsI(Na) Φ25 mm × 50 mm crystal with different exposure times in a 25% relative humidity environment under 662 keV γ-ray excitation of ^137^Cs.

Time (h)	0	3	6	9	12	24	27	30	33	36
Energy resolution (%)	11.26	11.19	10.59	11.12	11.76	10.84	10.79	10.54	10.96	11.11

**Table 2:** The adsorption energy of the CsI and CsI(Na) crystals.

Material	Adsorption Energy	R_O-H_	A_H-O-H_
CsI	0.1239 eV	0.973 Å	103.946°
CsI(Na)	−0.5344 eV	0.999 Å	105.111°
H_2_O	–	0.973 Å	103.946°

## Data Availability

Available on request.
